# Gold Nanoparticle-Based Hydrogel: Application in Anticancer Drug Delivery and Wound Healing In Vitro

**DOI:** 10.3390/pharmaceutics17050633

**Published:** 2025-05-09

**Authors:** Varshan Gounden, Moganavelli Singh

**Affiliations:** Nano-Gene and Drug Delivery Laboratory, Discipline of Biochemistry, University of KwaZulu-Natal, Private Bag X54001, Durban 4000, South Africa; 219006317@stu.ukzn.ac.za

**Keywords:** anticancer, wound healing, hydrogel, chitosan, gold nanoparticles, 5-fluorouracil, cytotoxicity

## Abstract

**Background/Objectives**: Due to the challenges faced by anticancer therapeutics, such as poor selectivity and metabolic degradation, novel delivery systems are needed to mitigate the adverse effects of chemotherapy. The management of chronic wounds is often overlooked and affects patients mentally and physically. The application of hydrogels can reduce deficiencies in drug delivery and wound healing due to their similarity to the extracellular matrix and stimuli-responsive properties. **Methods**: A chitosan (CS) hydrogel, cross-linked to gold nanoparticles (AuNPs), followed by the encapsulation of 5-fluorouracil (5-FU), was formulated. The physicochemical properties, drug release profiles, cytotoxicity, and wound healing in vitro were analyzed. **Results**: Fourier transform infrared spectroscopy and a UV-visible peak at 530 nm confirmed their successful synthesis. Transmission electron microscopy revealed spherical NPs of 89.31 nm, while scanning electron microscopy confirmed the porous network surface of the hydrogels. The thermogravimetric analysis demonstrated enhanced stability for the CS-Au hydrogel, while a non-Newtonian shear-thinning property was evident from rheology. Drug release showed a sustained, pH-dependent release with specificity for the acidic cancer microenvironment. The cytotoxicity assay demonstrated a specificity of the CS-Au-5-FU hydrogel for the cancer cells (HeLa and MCF-7) and diminished cytotoxicity in the non-cancer cells (HEK293). The scratch assay illustrated a complete closure of the wounds in HEK293 cells at low concentrations (15.63 and 31.25 µg/mL). **Conclusions**: The positive findings from this study confirm the potential of these CS-Au hydrogels to function as smart in vitro delivery systems and scaffolds for wound healing, warranting additional optimizations and in vivo studies.

## 1. Introduction

Cancer is characterized by the uncontrolled proliferation of cells within the body, which then metastasize to other tissues and organs, driven by key hallmarks including diminished growth restriction, suppression of the immune system, angiogenesis, resistance to apoptosis, and genetic instability. These cancer formation pathways directly disrupt fundamental physiological processes, resulting in mortality [1]. Significant progress in cancer identification and treatment has led to the creation of novel methodologies; nonetheless, chemotherapy continues to be the predominant strategy in cancer therapy. However, further improvements in chemotherapy are necessary to reduce the adverse effects due to poor administration techniques, multidrug resistance, and a lack of therapeutic specificity for the cancer tissue. 5-Fluorouracil (5-FU) is a frequently used chemotherapeutic agent, but it is rapidly metabolized in the body and has a restricted ability to selectively target cancer cells. This has led to severe side effects due to healthy cells being affected [2]. Hence, it is essential to develop advanced techniques for 5-FU administration to overcome its limitations and improve its efficacy.

Hydrogels are polymers characterized by hydrophilic attributes that undergo cross--linking to form three-dimensional networks. These networks can enlarge and absorb water many times their dry mass. They exhibit exceptional biocompatibility and can efficiently encapsulate hydrophilic drugs, owing to an elevated water content [3]. The phenomenon of ‘smart’ hydrogels undergoing a conformational change in response to stimuli has been extensively studied to facilitate regulated drug release [4]. The tumor microenvironment (TME) may serve as an area of interest for therapies that exploit the acidic characteristics of cancer cells attributed to acidosis. Hence, pH-sensitive hydrogels containing chitosan (CS) can be utilized for drug release in the acidic TME [5]. The therapeutic significance of CS is established by the abundance of positively charged amino groups. CS hydrogels exhibit swelling in the TME due to the protonation of amino groups at low pH. Due to the expansion of the pores between the intermolecular polymeric chains, biological fluid infiltrates the hydrogel, causing it to swell upon contact with the acidic TME. This releases the drug over time, specifically targeting cancer cells [6]. Several shortcomings, such as inadequate mechanical strength and dissolution in water, can be addressed by cross-linking.

Physical cross-linking is a suitable approach for synthesizing hydrogels, as it creates a three-dimensional reversible network by forming non-covalent bonds, thereby improving mechanical strength and enabling injectability. Metal nanoparticles (NPs) are ideal physical cross-linkers, as they provide additional mechanical support. Gold NPs (AuNPs) are non-toxic and biocompatible, providing a foundation for integration within a hydrogel [7]. The CS-Au hydrogel containing encapsulated 5-FU is an innovative treatment strategy, integrating the properties of AuNPs with CS hydrogels to enable the sustained release of 5-FU and mitigate adverse effects on patients.

While cancer is a major global scourge, the predicament of chronic and acute wounds has also impeded the social and economic climate, and much-needed change in healthcare is required to rectify these detrimental effects. Wound healing, which is an organized process, is disrupted during diseased states. The current wound-healing strategies have shown shortcomings in reducing the time needed for recovery. Hence, a new and improved system is required to overcome these challenges. Hydrogels have been presented as adequate wound dressings for chronic and acute wounds through their ideal properties, such as maintaining a moist environment, absorption of exudates and necrotic tissue, and flexibility in shape to cover wounds with different morphologies [8]. Hydrogels consist of natural or synthetic polymers. Synthetic polymers exhibit stability and convenience but lack biocompatibility, making natural polymers the preferred choice because their composition and properties closely resemble the fundamental features of bodily tissues [9].

CS, a natural polymer, is widely used in hydrogel formulations for wound healing, owing to its beneficial attributes. CS resembles glycosaminoglycans in the extracellular matrix (ECM), and its cationic nature contributes to its antibacterial properties [10]. Developing nanogels or NP-cross-linked hydrogels has paved the way for enhanced wound treatments. Nanogels have attracted attention for their capacity to combine the physical attributes and increased water absorption properties of hydrogels with the tunable size and shape of nanomaterials to conjugate drugs and biomolecules [11]. Gold nanogels have been sought after due to their inherent low toxicity, antibacterial properties through membrane disruption, increased stability, and photothermal potential due to their surface plasmon resonance (SPR) effect [12].

The current study is focused on the in situ physical cross-linking of a CS hydrogel with AuNPs. This novel synergistic hydrogel may provide a dual purpose in drug delivery and wound healing by serving as a potential solution for reducing the duration of wound healing, improving overall patient health, and reducing the load borne by the current healthcare system.

## 2. Materials and Methods

### 2.1. Materials

Chitosan (CS) (25 kDa, 75% deacetylated), acrylic acid (C_3_H_4_O_2_), 5-fluorouracil (5-FU) (MW = 130.08 g/mol), and lysozyme (≥90% proteins, activity ≥ 40 000 U/mg) were sourced from Sigma-Aldrich, St. Louis, MO, USA. Gold (III) chloride trihydrate (99%) (HAuCl_4_⋅3H_2_O), 3-[4,5-dimethylthiazol2-yl]-2,5-diphenyl tetrazolium bromide (MTT), phosphate-buffered saline (PBS), and dimethyl sulfoxide (DMSO) were procured through Merck, Darmstadt, Germany. Human cervical carcinoma (HeLa), breast adenocarcinoma (MCF-7), and embryonic kidney (HEK293) cells were initially supplied by the ATCC, Manassas, VA, USA. Eagle’s minimum essential medium (EMEM) with L-glutamine, penicillin/streptomycin/amphotericin B (100×) antibiotic mixture [amphotericin B (25 μg·mL^−1^), penicillin (10,000 U·mL^−1^), and streptomycin sulfate (10,000 μg·mL^−1^)] and trypsin–versene mixture were procured from Lonza Bio Whittaker, Walkersville, MD, USA. Fetal bovine serum (FBS) was sourced from Hyclone, GE Healthcare, Salt Lake City, UT, USA. Sterile plasticware was purchased from Corning Inc., Corning, NY, USA. Ultrapure (18 MOhm) water (Milli-Q Academic, Millipore, Guyancourt, France) was utilized in preparations, and all the reagents were of analytical quality.

### 2.2. Synthesis of Chitosan–Gold (CS-Au) Hydrogel

**Figure 1:**
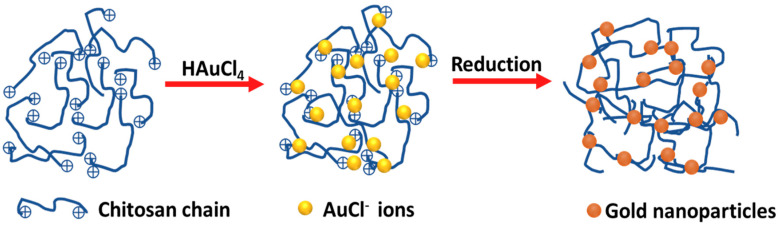
Schematic representation for the formation of gold nanoparticles in the cross-linked chitosan hydrogel.

A solution was made by dissolving 0.60 g of CS in 0.3 mL of acrylic acid (1.048–1.050 g/mL) and 20 mL of deionized water. Upon complete dissolution of the CS, the solution was heated to 70 °C, followed by the injection of 1% (*w*/*v*) HAuCl_4_ (1 mL). A yellowish-brown hydrogel was immediately produced. After continuous stirring for 10 min with heating, the color of the hydrogel changed to a wine red. The resultant CS-Au hydrogel was cooled to room temperature. Figure 1 provides a simple diagram illustrating the synthesis process.

### 2.3. UV-Visible (UV-Vis) and Fourier Transform Infrared (FTIR) Spectroscopy

The absorption spectrum of the CS-Au hydrogel was obtained at 1 nm intervals within a wavelength range of 400–650 nm using a UV-vis spectrophotometer (Jasco V-730 Bio Spectrophotometer, JASCO Corporation, Hachioji, Japan). The maximum absorption peak was referenced against the literature.

FTIR was utilized to confirm the specific functional groups present in the components of the hydrogels. The samples were freeze-dried and analyzed at 25 °C. The analysis was performed using a Perkin Elmer Spectrum 100 FTIR spectrometer (Waltham, MA, USA), which was fitted with a Universal Attenuated Total Reflectance (ATR) unit. The wavelength range used for the analysis was 400–4000 cm^−1^. The distinctive peaks of the samples were verified by comparing them to those documented in the literature.

### 2.4. Zeta Potential Analysis

The zeta potentials of the hydrogels were determined by employing dynamic light scattering (DLS) in a Malvern Zetasizer Nano-ZS instrument (Malvern Instruments Ltd., Malvern, UK). The experiments were performed at ambient temperature using DTS0012 polystyrene cuvettes (Malvern Panalytical, Malvern, UK). The samples were diluted (1:500 *v*/*v*) in water, and the measurements were conducted in triplicate.

### 2.5. Transmission Electron Microscopy (TEM)

The morphology and size of the AuNPs embedded in the hydrogels were analyzed using transmission electron microscopy (TEM) in their dry state. Approximately 10 µL of each sample was sonicated for 15 min and then placed on a 400-mesh carbon-coated copper grid (Ted Pella Inc., Redding, CA, USA). After air-drying for 1 h, the samples were examined under a JEOL 1400 transmission electron microscope (Jeol, Tokyo, Japan). A 3-megapixel digital camera positioned on the side of the iTEM Soft Imaging Systems (SIS) Megaview III was utilized to capture and process the images of the hydrogels.

### 2.6. Scanning Electron Microscopy (SEM)

The surface morphology of the hydrogels was examined using scanning electron microscopy (SEM). The ZEISS Ultra Plus FEGSE microscope with SmartSEM software V6 (Carl Zeiss, Oberkochen, Baden-Württemberg, Germany) was used to acquire images. The hydrogels were mounted onto aluminum stubs with double-sided carbon tape. The samples were gold-coated using a gold sputter coater (Quorum Q150 RES, Lewes, UK) and then viewed and imaged.

### 2.7. Thermogravimetric Analysis (TGA)

The thermal characteristics of the hydrogels were analyzed using the Netzsch DSC-200 PC Phox differential scanning calorimetry (DSC) (Selb, Germany). The DSC-TGA assessment of the hydrogels was conducted using a closed alumina crucible. The analysis was undertaken in a temperature range of 25–650 °C, with a heating rate of 10 °C/min, in an atmosphere of nitrogen.

### 2.8. Rheological Studies

The viscosity and thixotropy of the hydrogels were measured using an Anton Paar Modular Compact Rheometer 302 (Anton Paar GmbH, Graz, Austria) at room temperature. Measurements were conducted using an LV spindle 64 (Ametek Brookfield, Middleboro, MA, USA). The viscosity measurements, expressed in centipoise (cP), were obtained at several shear rates, specifically 10, 20, 50, 60, and 100 rpm, using a torque setting range of 10 to 100%. The viscometry values were determined in triplicate.

### 2.9. Reswelling and pH Sensitivity

The equilibrium swelling ratio of the CS-Au hydrogel at pH 4.5, 7.4, and 10.5 was assessed using a conventional weight measurement technique. Initially, the dehydrated hydrogel was submerged in distilled water at room temperature and left for 24 h to achieve an equilibrium swelling state. Subsequently, the hydrogel was removed, and the surface was dried using filter paper. The equilibrium swelling ratio (SR) was determined using Equation (1).

(1) $$\mathrm{SR}=\frac{Wwet}{Wdry}\times 100\%$$

Wwet and Wdry represent the hydrogel weights obtained before and after the swelling test.

### 2.10. Drug Encapsulation

A vial containing 15 mg of freeze-dried hydrogel was immersed in 5 mL of distilled water. The hydrogel was then soaked in a 3 mg/mL 5-FU solution. The vials were kept at room temperature for 2 days to allow the hydrogel to reach a state of equilibrium. The 5-FU content was determined using UV-vis spectroscopy at a wavelength of 266 nm. All procedures were conducted in the dark. Equation (2) was used to calculate the encapsulation efficiency of 5-FU.

(2) $$\mathrm{Encapsulation}\;\mathrm{Efficiency}\;(\%)=\frac{Total\left(5\text{-}FU\right)-(5\text{-}FU)\;in\;vial}{Total\;5\text{-}FU}\times 100\%$$

### 2.11. In Vitro Degradation

The gravimetric analysis measured the degree of in vitro degradation for the CS-Au and CS-Au-5-FU hydrogels, which were cured for 2 h at 37 °C. The brief curing time was chosen to analyze the hydrogels in settings that closely resemble those in an in vivo environment. All samples were maintained in solid form at the start of the testing. The hydrogels, weighing (240 mg), were subjected to 24 h incubation in PBS at 37 °C until equilibrium was reached. The total weight of the samples was then measured over 28 days in 10 mL of PBS containing lysozyme (1.5 μg/mL) at 37 °C. The enzyme concentration employed was comparable to that observed in human serum. The lysozyme was replenished every 2 days to mimic uninterrupted enzyme activity. At predetermined periods, hydrogels were extracted from the solution and dried on filter paper to eliminate surface moisture, and the weight was measured. Equation (3) was used to express the degree of in vitro degradation as a percentage of mass loss.

(3) $$\mathrm{Weight}\;\mathrm{loss}\;\%=\frac{wi-wt}{wi}\times 100\%$$

where wi and wt denote the initial and final weights of the hydrogel. The assay was performed in triplicate.

### 2.12. Drug Release

The drug release from the CS-Au-5-FU hydrogel was quantified over a 48 h timeframe in PBS adjusted to pH 4.5, 6.5, and 7.4, respectively, allowing for the assessment of the pH-responsive properties of the hydrogel. Approximately 15 mg of the freeze-dried drug-loaded hydrogel was placed in 5 mL of PBS at the respective pH levels. The experiment was conducted at 37 °C, over 48 h. At intervals of 4 h, 10 µL aliquots of the dialysates were removed and replaced with the same volume of fresh PBS to maintain the sink volume. The drug quantity in the dialysate was determined using UV-vis spectroscopy at a wavelength of 266 nm. The measurements were conducted in triplicate for each sample. The amount of 5-FU released was determined using Equation (4).

(4) $$\mathrm{Cumulative}\;\mathrm{drug}\;\mathrm{release}\;(\%)=\frac{Absorbance\;of\;free\;5\text{-}FU}{Absorbance\;of\;total\;5\text{-}FU}\times 100\%$$

### 2.13. Drug Release Kinetics

To examine the drug release mechanism from the CS-Au hydrogel, release results were evaluated utilizing the following models:

Zero order: f1 = K_0_t. (5)

First order: f1 = 1 − e-K1.t(6)

Korsmeyer-Peppas model: f1 = Mi/M∞ = Ktn(7)

The symbols f1 and Mi/M∞ represent the drug’s fractional release at time t, K denotes the rate constant, and n signifies the diffusion exponent.

The kinetic models were chosen to align with the drug release data, and conclusions were drawn using the coefficient of determination (r^2^) values.

### 2.14. Cell Culture and Maintenance

The cell-based assays were performed in an Airvolution Class II biosafety laminar flow hood. The cells were grown in 25 cm^2^ tissue culture flasks containing 5 mL of EMEM, enriched with 10% (*v*/*v*) FBS and 1% antibiotics (100 U/mL penicillin, 100 μg/mL streptomycin). The cells were maintained at 37 °C in a HEPA-class 100 Steri-Cult CO_2_ incubator (Thermo-Fisher Corporation, Waltham, MA, USA). Confluent cells were plated into multi-well plates for subsequent experiments.

### 2.15. In Vitro Cytotoxicity

The HEK293, HeLa, and MCF-7 cells were seeded in 48-well plates at a density of 3.5 × 10^4^ cells per well. After a 24 h incubation, the medium was removed from each well and replenished with 200 µL of fresh medium. The CS, CS-Au hydrogels and free 5-FU solutions were added to the cells at varying concentrations (10, 20, 30, 40, and 50 µg/mL). The assay was conducted in triplicate. Cells were incubated for 48 h at 37 °C. Thereafter, the medium was substituted with 200 µL of complete medium containing 20 µL of MTT solution (5 mg/mL in PBS). The cells were then incubated for 4 h at 37 °C. After the MTT-medium mixtures were removed, 200 µL of DMSO was added to dissolve the resulting purple formazan crystals. The absorbances were measured at a wavelength of 540 nm using a Mindray MR-96A microplate reader (Vacutec, Hamburg, Germany), with DMSO serving as the blank. The cell viability was determined using Equation (8).

(8) $$\mathrm{Cell}\;\mathrm{Viability}\;(\%)=\frac{Absorbance\;of\;treated\;cells}{Absorbance\;of\;control\;cells}\times 100\%$$

### 2.16. Scratch Assay

A scratch wound assay was used to measure the growth and migration of the HEK293 cells. The cells were seeded on a 48-well plate with a density of 1.3 × 10^5^ cells per well and incubated at 37 °C for 24 h. A linear wound was created in the monolayer with a sterile pipette tip. The cells were exposed to 15.63, 31.25, 62.5, and 125 µg/mL of the CS and CS-Au hydrogels. The width of the scratched region was measured at three distinct sites to observe cell proliferation directly following the scratch and after 24 h. Images were obtained using a Nikon inverted phase contrast microscope (Nikon, Tokyo, Japan). Wound closure was calculated using Equation (9).

(9) $$\mathrm{Wound}\;\mathrm{closure}=\frac{Wound\;area\;at\;day\;0-Wound\;area\;at\;day\;1}{Wound\;area\;at\;day\;0}\times 100\%$$

### 2.17. Statistical Analysis

The data have been reported as the mean ± standard deviation (±SD, n = 3). The FTIR spectra were generated using the Origin 2019b software. Statistical analysis of the MTT assay was conducted using one-way ANOVA and Dunnett’s post hoc test. The tests were considered statistically significant at *p* < 0.01 and *p* < 0.05. Every experimental value was compared with its corresponding control. The software utilized was Microsoft Excel 2024 (Microsoft, Redmond, WA, USA) and GraphPad Prism 9 (GraphPad Software Inc., Boston, MA, USA).

## 3. Results

### 3.1. UV-Vis and FTIR Spectroscopy

**Figure 2:**
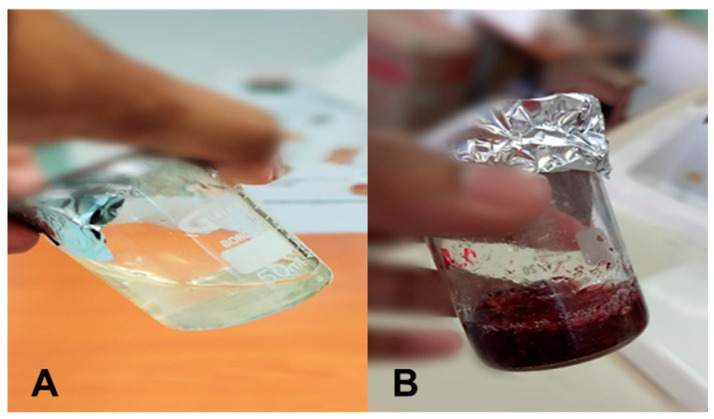
Images of the formulated (**A**) CS and (**B**) CS-Au hydrogels showing the viscosity and color change.

The CS hydrogel containing AuNPs (CS-Au) visually demonstrated the effective reduction of HAuCl_4_·3H_2_O by transitioning to its characteristic wine-red color (Figure 2). When inclined, the hydrogel’s resistance to flow indicated mechanical stability and increased viscosity.

**Figure 3:**
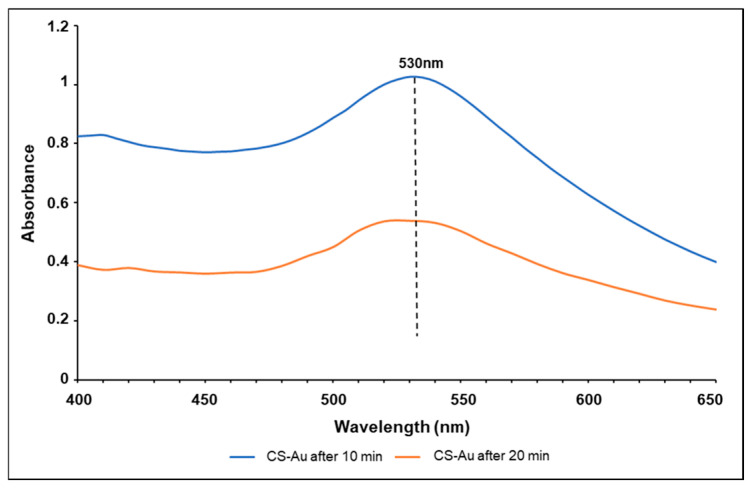
UV-vis spectroscopy of the CS-Au hydrogel after heating for 10 and 20 min.

The UV-vis spectra for the CS-Au hydrogels after heating for 10 and 20 min are shown in Figure 3. The SPR of the AuNPs showed a singular narrow peak at 530 nm, aligning with the predicted λmax of AuNPs, presenting tangible confirmation of the successful synthesis of the AuNPs [13]. The peak singularity indicates negligible by-product formation. A significant reduction in absorbance intensity was apparent after 10 min of heating.

**Figure 4:**
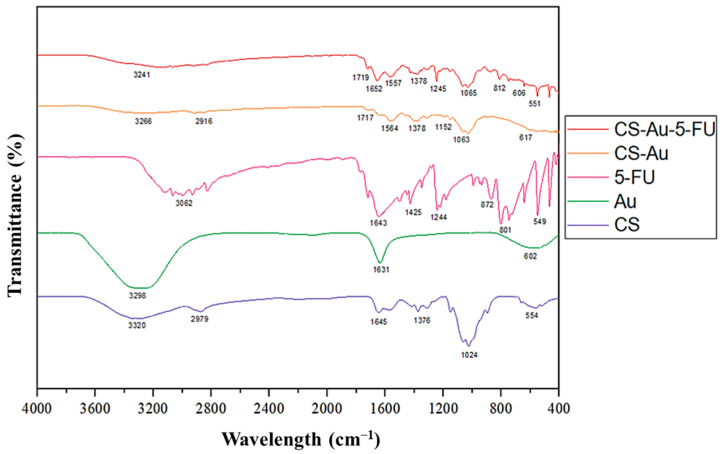
FTIR spectra of CS, AuNP, 5-FU, CS-Au, and CS-Au-5-FU.

The successful formulation of the CS-Au hydrogel was corroborated by the FTIR spectra (Figure 4), exhibiting corresponding peaks in the AuNPs and CS hydrogel. The emergence of the asymmetrical carboxylate stretching band at 1557 cm^−1^ confirmed the synthesis of AuNPs [14]. The peak at 3241 cm^−1^ indicated N-H and O-H stretching, while peaks at 2916 cm^−1^, 1652 cm^−1^, and 1063 cm^−1^ corresponded to the C-H symmetric stretching, primary amine N-H bending, and C-O stretching, respectively [15]. In contrast to the standard CS hydrogel, slight peak changes and an enhancement in the strength of the amine peak suggested effective AuNP cross-linking. The characteristic pyrimidine peak was visible in the 5-FU spectra at 1245 cm^−1^. The vibration of the imide stretch may also be observed at peaks 812 cm^−1^ and 551cm^−1^ [16]. The decrease in strength of the characteristic 5-FU peaks in the CS-Au-5-FU verified the validity of the encapsulated drug. The peaks indicate a notable alteration in the degree of chemical interactions and corroborate the drug encapsulation.

### 3.2. Zeta Potential (ZP) Analyses

The ZP for the CS-Au and CS-Au-5-FU hydrogels were 11.1 ± 0.1 mV and 15.87 ± 1.18 mV (Table 1), respectively, indicating that both hydrogels may offer short-term stability. However, CS may act as a high-molecular-weight stabilizer through steric stabilization. Therefore, values that approximate around ±20 mV or lower can provide sufficient stabilization. Large polymers may shift the plane of shear further from the particle surface, thus reducing the ZP. Hence, these particles may exhibit a high charge and stability but record a low ZP [17].

### 3.3. Transmission Electron Microscopy (TEM)

**Figure 5:**
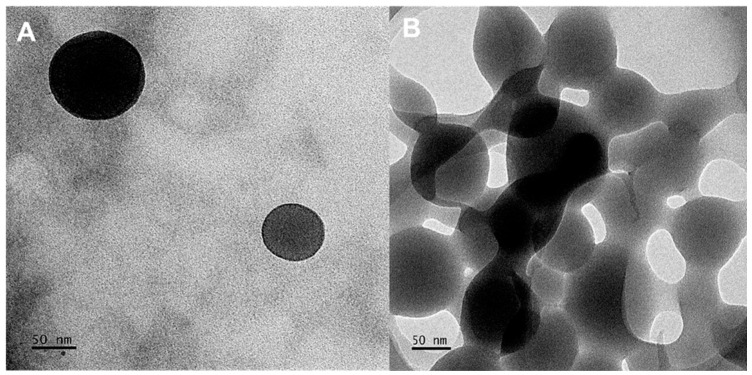
TEM images of the CS-Au hydrogel exhibiting a (**A**) spherical morphology and (**B**) a cross-linking matrix.

TEM utilizes a beam of electrons to examine the homogeneity, ultrastructural morphology, and dispersion of NPs. The CS-Au hydrogels (Figure 5) exhibited a smooth spherical morphology characterized by a consistent size and shape distribution, with a diameter of approximately 89.31 nm. The diameters of the NPs observed by TEM (Figure 5A) ranged from 10 to 200 nm, rendering them appropriate for drug delivery as they can penetrate biological barriers and avoid clearance. Figure 5B illustrates the physical cross-linking matrix of the AuNPs, which underpins enhanced mechanical stability and rigidity.

### 3.4. Scanning Electron Microscopy (SEM)

**Figure 6:**
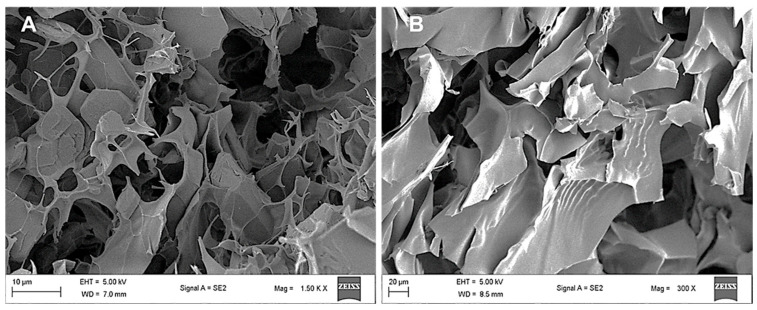
SEM images depicting the surface morphology of the (**A**) CS and (**B**) CS-Au hydrogels.

The morphological characteristics of the CS and CS-Au hydrogels were also observed by SEM (Figure 6). SEM elucidated the porosity and properties of the hydrogel framework. In addition, the influence of cross-linking on pore size was observed (Figure 5B). The freeze-dried CS (Figure 6A) and CS-Au (Figure 6B) hydrogels were analyzed for surface and cross-sectional morphology, revealing a porous matrix with an interconnected channel-like framework. The pore sizes are irregular, ranging from 5 to 20 µm for both hydrogels; however, it is noticeable that the CS-Au hydrogel exhibits smaller yet more structured pore sizes. The cross-linking of the CS-Au hydrogel creates a more tightly packed network, hence a reduction in pore size. Due to their internalization into the hydrogel matrices, AuNPs are not visible on the hydrogel surfaces in the SEM images. The interconnected pores enabled the freeze-dried hydrogels to undergo significant swelling, as these channel-like structures facilitate water absorption in the gel matrix by capillary movement, resulting in rapid solvent transport in the matrix.

### 3.5. Thermogravimetric Analysis (TGA)

**Figure 7:**
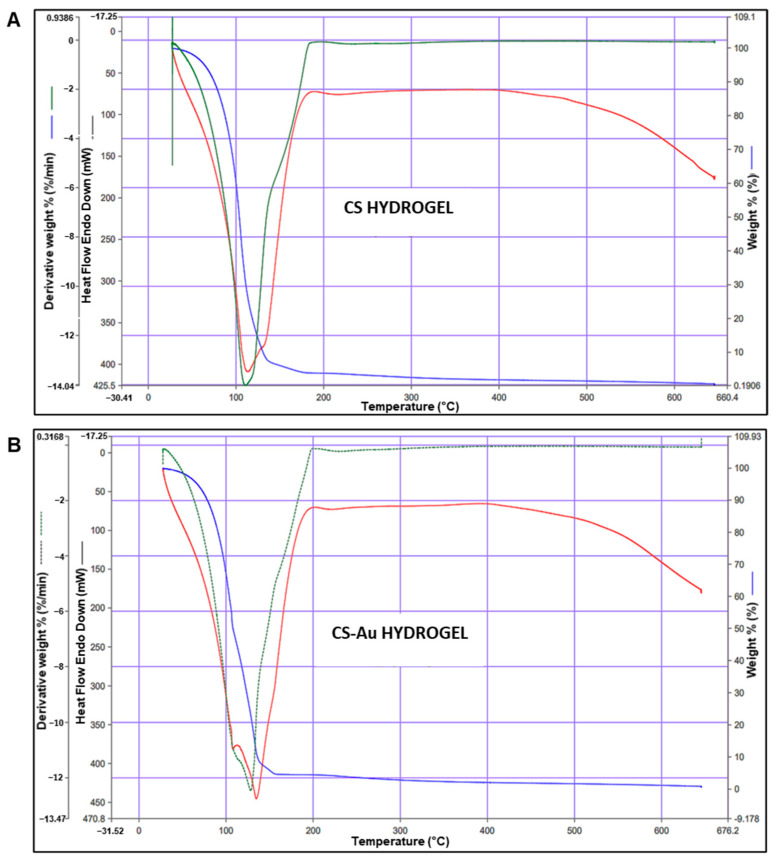
Thermogravimetric analysis of the (**A**) CS and (**B**) CS-Au hydrogels. The blue lines represent the weight, the green lines the derivative weight%, and the red line represents the differential scanning calorimetry of each hydrogel.

TGA is often used to assess the thermal stability of hydrogels. Sample mass fluctuations are monitored relative to temperature, rising at a constant rate of 1–10 °C/min. The initial mass loss stage is identified by a reduction in weight below 100 °C. The weight loss percentages for the CS (Figure 7A) and CS-Au (Figure 7B) hydrogels were 39.95% and 32.94%, respectively. This phase typically entails the evaporation of water and other chemically unstable substances adsorbed on the hydrogel surface. In the CS hydrogels, this refers to the loss of water absorbed from the polysaccharide framework, which comprises hydrophilic hydroxyl and amino groups. At 37 °C, the weight percentages were 99.46% for the CS and 99.54% for the CS-Au hydrogel.

The subsequent phase is the decomposition stage (100–600 °C), during which most of the weight loss occurs due to the degradation of the CS polymer framework. In addition, stability is further evidenced by the temperature of the endothermic peak. The CS hydrogel has an endothermic peak at 113.61 °C, whereas the CS-Au hydrogel demonstrated an endothermic peak at 134.82 °C. These findings are consistent with the literature [18].

### 3.6. Rheological Studies

**Figure 8:**
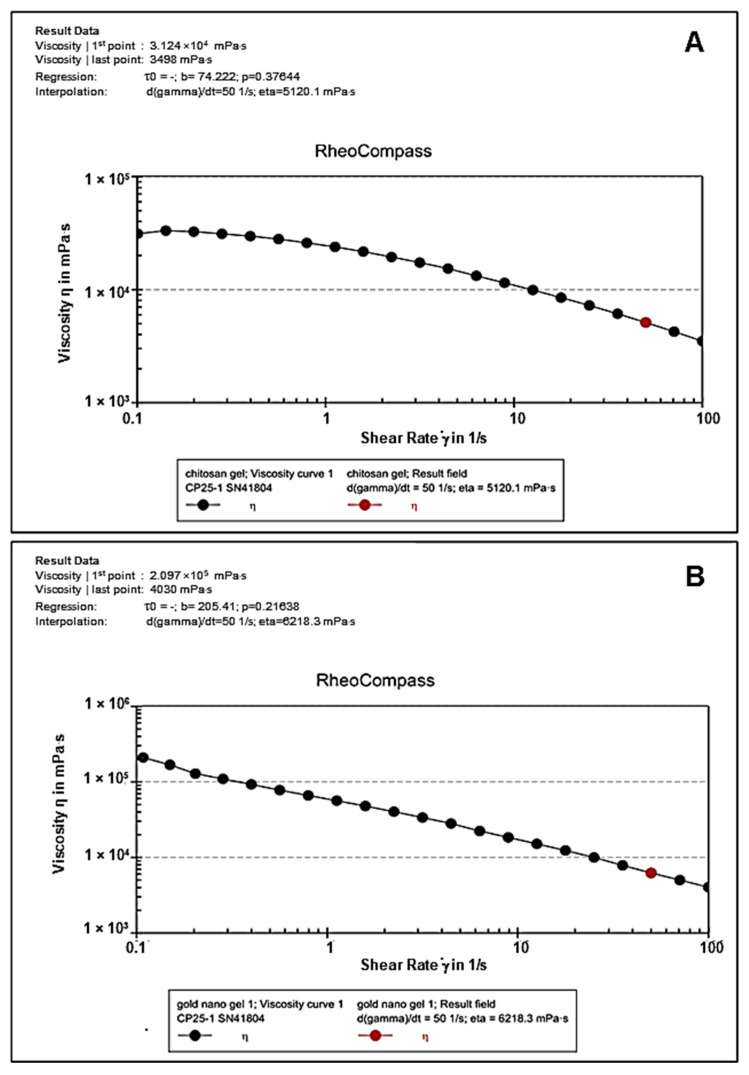
Flow curves depicting viscosity vs. shear rate of the (**A**) CS and (**B**) CS-Au hydrogels.

Rheology is the preferred approach for characterizing the dynamic and static viscoelastic behavior of hydrogels. The CS (Figure 8A) and CS-Au (Figure 8B) hydrogels exhibited shear-thinning activity. Over a shear rate of 0.1 to 100 (1/s), the CS and CS-Au hydrogels showed a decrease in viscosity of 3.124 × 10^0.4^ mPa/s to 3498 mPa/s and 2.097 × 10^0.5^ mPa/s to 4030 mPa/s, respectively. Hence, these hydrogels exhibited enhanced deformability through needles and catheters, facilitating the delivery of drugs by injection. The disparity in the initial viscosities of the hydrogels arises from the AuNP cross-linking matrix in the CS-Au hydrogel, which increases the initial viscosity and enhances rigidity.

**Figure 9:**
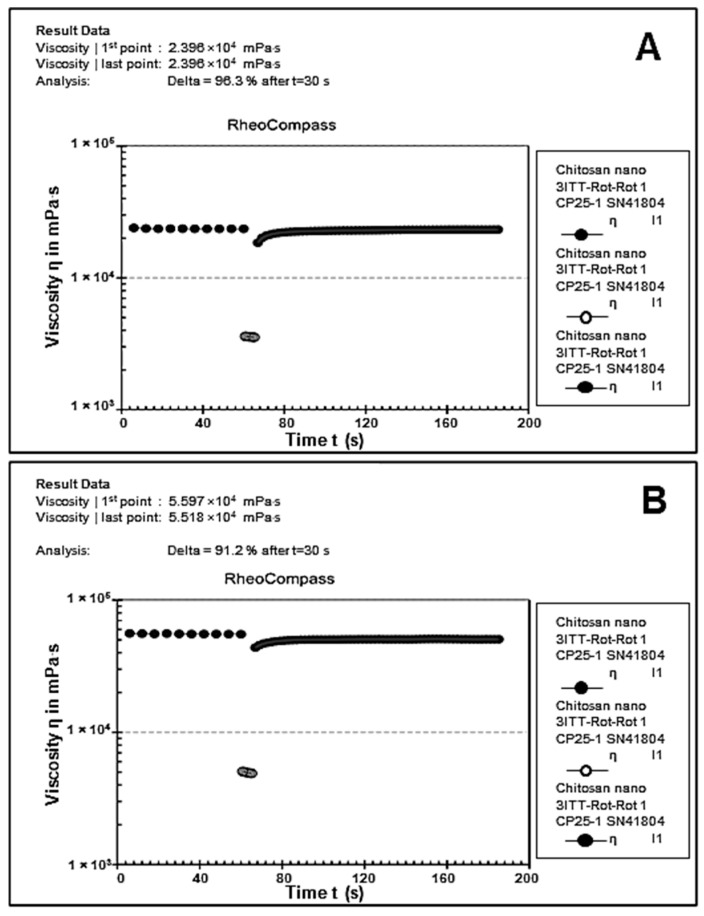
Thixotropy study demonstrating self-healing for the (**A**) CS and (**B**) CS-Au hydrogels.

The thixotropic effect on the CS and CS-Au hydrogels is illustrated in Figure 9. Thixotropy is the ability of specific fluids and hydrogels to decrease in viscosity when subjected to constant shear and then return to their initial viscosity once the force is removed within an appropriate timeframe (self-healing). The relative viscosities of the CS and CS-Au hydrogels before shear application were 2.396 × 10^0.4^ mPa/s (Figure 9A) and 5.597 × 10^0.4^ mPa/s (Figure 9B), respectively. Shear force was applied between 60 and 65 s, resulting in a viscosity reduction measured at 3543 mPa/s for the CS hydrogel and 4898.7 mPa/s for the CS-Au hydrogel. Following shear stress, a viscosity recovery of 2.358 × 10^0.4^ mPa/s and 5.518 × 10^0.4^ mPa/s was observed within 30 s for the CS and CS-Au hydrogels, respectively. The recovery in viscosity of the hydrogels indicated a self-healing property following injection.

### 3.7. Re-Swelling and pH Sensitivity

**Figure 10:**
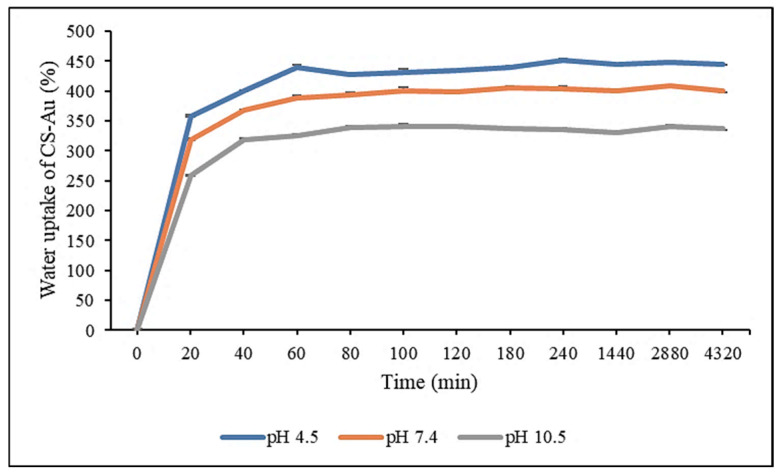
Re-swelling and pH sensitivity studies of the CS-Au hydrogel at pH 4.5, 7.4, and 10.5. Triplicates of each hydrogel were analyzed, and each point represents the mean value ± standard deviation.

The swelling capacity of the CS-Au hydrogel was assessed using the equilibrium SR in distilled water at room temperature. The study was conducted at pH 4.5, 7.4, and 10.5 to evaluate the pH sensitivity of the hydrogel and its expansion and water absorption when submerged in the various pH solutions. After 20 min, rapid pH-dependent water uptake was observed for the freeze-dried CS-Au hydrogel with swelling ratios of 357.16, 318.57, and 259.1% at pH 4.5, 7.4, and 10.5, respectively (Figure 10). Equilibrium was achieved after 1 h, with the swelling ratios reaching 439.93, 388.23, and 325.87% at pH 4.5, 7.4, and 10.5, respectively. The swelling maxima recorded for the CS-Au hydrogels were 450.77, 409.07, and 340.83 at pH 4.5, 7.4, and 10.5, respectively. The CS-Au hydrogel displayed notable pH dependence, with markedly enhanced water absorption in acidic solutions relative to basic solutions and at physiological pH (Figure 10).

### 3.8. Drug Encapsulation

The concentration of 5-FU in the solvent following immersion of the freeze-dried CS-Au hydrogel was analyzed using UV-vis spectroscopy to determine the encapsulation efficiency of 5-FU. A favorable EE of 77.71% for the CS-Au hydrogel was achieved.

### 3.9. In Vitro Degradation

**Figure 11:**
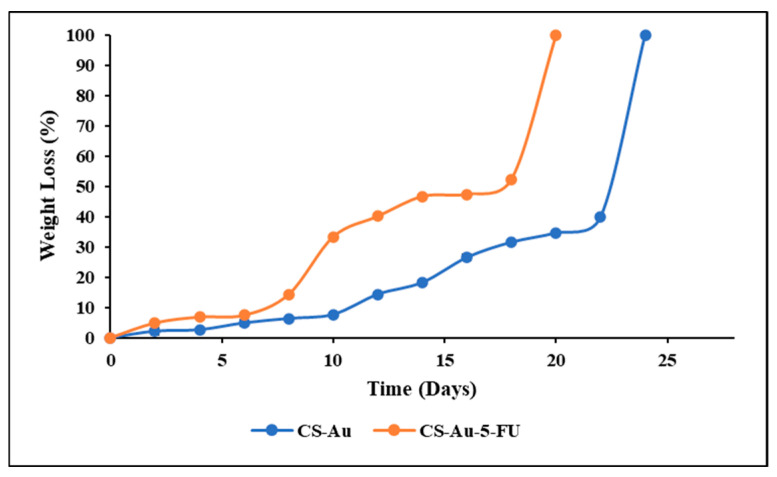
Comparison of the weight loss of the CS-Au and CS-Au-5-FU hydrogels as a function of incubation time in PBS containing 1.5 μg of lysozyme/cm^3^ at 37 °C.

The in vitro degradation was assessed by measuring the hydrogels’ mass loss over time in a simulated physiological environment. Lysozyme was incorporated into a PBS solution to simulate the in vivo degradation capabilities. Figure 11 shows that both CS hydrogels cross-linked with AuNPs and encapsulated with 5-FU exhibited breakdown by lysozyme and comparable weight loss profiles. The standard CS-Au hydrogel showed a slightly slower degradation rate than the CS-Au-5-FU hydrogel. This may be due to the interference of 5-FU with the intermolecular interactions between the CS chains and AuNPs, resulting in a less compact network. The CS-Au-5-FU and CS-Au hydrogels both displayed gradual degradation. However, both hydrogels lost their structural integrity on Day 20 and Day 24, respectively.

### 3.10. Drug Release and Kinetics

**Figure 12:**
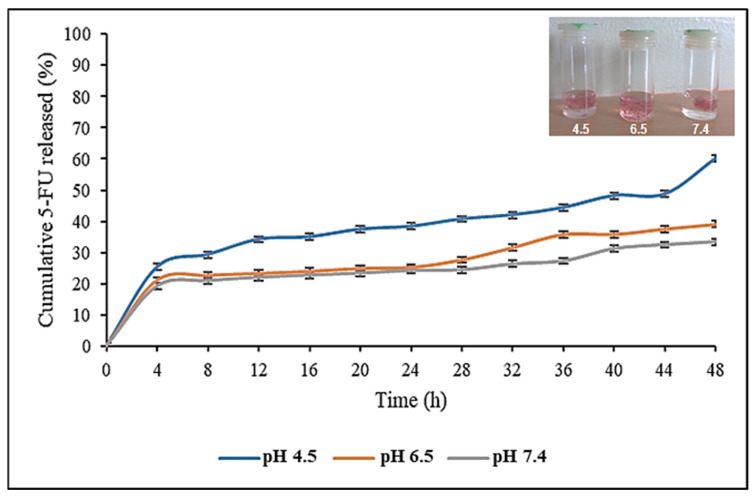
In vitro drug release profile of 5-FU from the CS-Au-5-FU hydrogel at pH 4.5, 6.5, and 7.4, at 37 °C. Triplicates of each hydrogel were analyzed, and each point represents the mean value ± standard deviation. The inset image shows the expansion of the hydrogel at pH 4.5, 6.5, and 7.4.

The pH-responsive drug release profile of the CS-Au-5-FU hydrogel in simulated in vivo conditions was evaluated at pH 4.5, 6.5, and 7.4 (Figure 12). Cancer tissue has a mildly acidic microenvironment (pH 4.0–5.5). Hence, the pH parameters were chosen to simulate the acidic cancer and near-neutral physiological environment. The CS-Au-5-FU exhibited an initial release of 5-FU after 4 h at all pH levels, ranging from 19% to 25%. A more sustained and controlled release was evident after this initial burst release. The release of 5-FU at pH 4.5 exhibited a biphasic pattern, with a secondary burst occurring after 44 h. After 48 h, 60.21% of 5-FU was released at a pH of 4.5, 39.04% at pH 6.5, and 33.41% at pH 7.4, respectively.

The hydrogels in acidic pH swelled significantly compared to those at pH 7.4 (Figure 12 inset). The drug release patterns of the hydrogel displayed labile behavior, suggesting that the release of 5-FU from the porous matrix was primarily regulated by the pH. This pH sensitivity would enable the drug to be released more effectively at the acidic tumor target sites. As a reduced amount of drug (33.41%) was released at physiological pH, the hydrogel offers promise for therapy by reducing cytotoxicity in healthy cells.

The in vitro drug release data were analyzed using three kinetic models (zero order, first order, and Korsmeyer–Peppas). The kinetic assessment of drug release (Table 2) suggests that the predominant release mechanism across all pH levels was via the Korsmeyer–Peppas model. This model was designed primarily for the release of drugs from a mesh of polymer chains, such as a hydrogel [19]. The Korsmeyer–Peppas model generalizes the drug delivery mechanism via the release exponent “n”, where n ≤ 0.5 indicates Fickian diffusion, n > 1 signifies non-Fickian diffusion, and 0.5 < n < 1 represents anomalous diffusion [20]. All values of “n” were below 0.5, suggesting that the release mechanism adhered to the principle of Fickian diffusion. In the Fickian model, the solvent transport or diffusion rate considerably exceeds the rate of the relaxation of polymer chains. In the swollen state, polymer chains exhibit significant mobility, facilitating solvent penetration.

### 3.11. In Vitro Cytotoxicity

**Figure 13:**
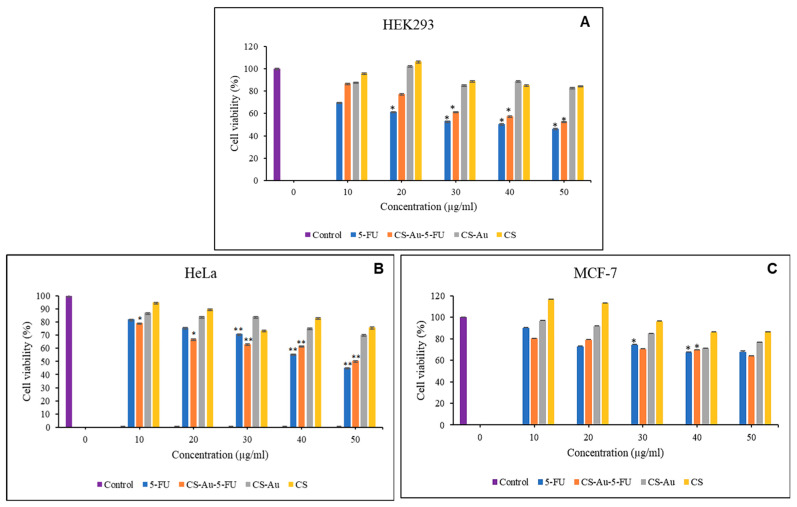
MTT cytotoxicity of the free 5-FU, CS-Au-5-FU, CS-Au, and CS hydrogels in (**A**) HEK293, (**B**) HeLa, and (**C**) MCF-7 cells. Data are represented as means ± SD (n = 3). * *p* < 0.05, ** *p* < 0.01 were considered statistically significant.

The cytotoxic effects of the CS, CS-Au, CS-Au-5-FU hydrogels, and free 5-FU on two cancer (HeLa and MCF-7) and one non-cancer (HEK293) cell line, were examined using the quantitative, colorimetric MTT assay. The fundamental principle posits that only metabolically active cells can sustain their mitochondrial enzyme function and convert the water-soluble, yellow tetrazolium salt into insoluble, purple formazan using its dehydrogenase enzymes [21,22]. The cytotoxicity profiles of the CS, CS-Au, CS-Au-5-FU hydrogels, and free 5-FU can be seen in Figure 13A–C.

The HEK293 cells were utilized as a control to assess the cytotoxic effects on non-cancer cells. Both CS and CS-Au hydrogels exhibited low cytotoxicity in the HEK293 cells. The cytotoxicity of CS and CS-Au is comparable at all concentrations, with the lowest being 82.76% for CS-Au at 50 µg/mL. The analogous results reflect the relative non-toxic nature of the AuNP cross-linkers, owing to their chemically inert core, which is further enhanced by CS functioning as a protective barrier in the CS-Au hydrogel. Cell proliferation is observed at a concentration of 20 µg/mL for both the CS and CS-Au hydrogels, with increased viabilities of 106.27% and 102.13%, respectively. This concentration may be optimal for the hydrated state of the hydrogels, which mimics the ECM and facilitates nutrient and oxygen transport through the pores.

The nanocomplex exhibited a concentration-dependent anticancer effect in the HeLa and MCF-7 cells (Figure 13B,C). The CS and CS-Au demonstrated minor anticancer effectiveness, showing moderate reductions in cell viability in a concentration-dependent manner, with a minimum cell viability of 70.24%. Hence, it may be concluded that the anticancer activity of the AuNPs makes them suitable as cross-linkers by enhancing the hydrogel’s potential in cancer therapy. The nanocomplex exhibited cytotoxicity that resembled that of free 5-FU, with negligible variation observed in the MCF-7 cells. In the HeLa cells (Figure 13B), a significant reduction in cell viability was observed at lower doses compared to the free 5-FU. However, at higher concentrations (40 and 50 µg), free 5-FU demonstrated greater cytotoxicity. Treatment with lower quantities of the nanocomplex is an advantageous alternative to free 5-FU, as it offers elevated cytotoxicity towards cancer cells while reducing cytotoxicity in non-cancer cells, hence limiting adverse side effects.

### 3.12. Scratch Assay

**Figure 14:**
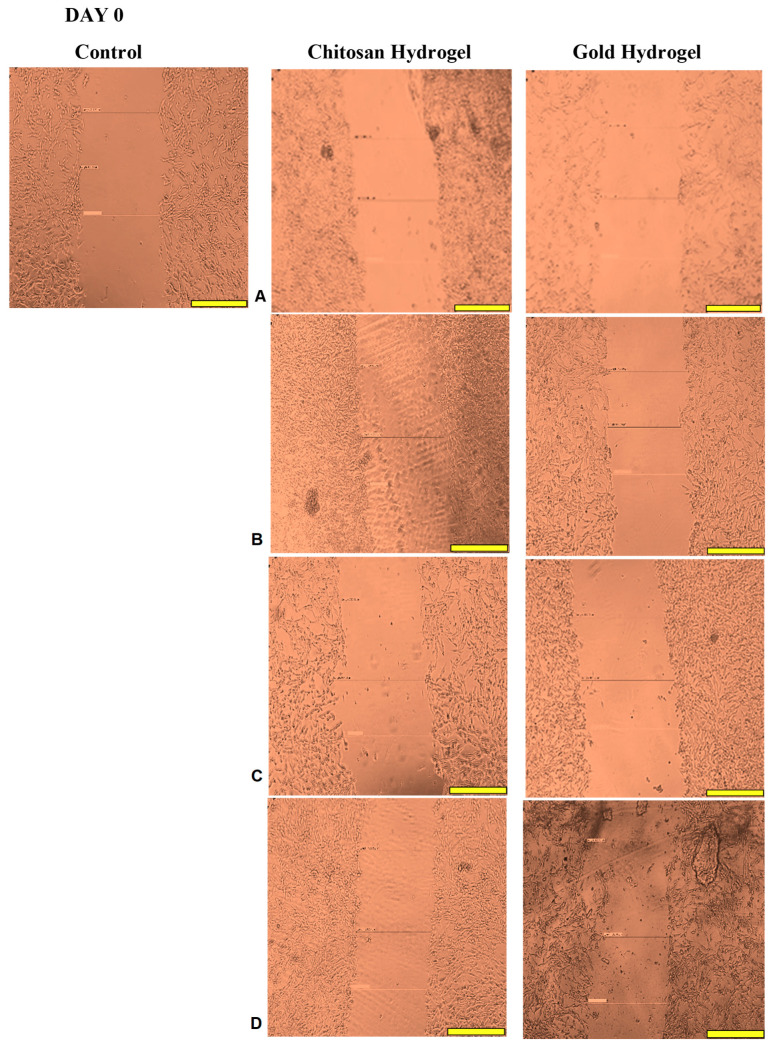
Images displaying the migration of HEK293 cells for wound closure when treated with (**A**) (15.63 µg/mL), (**B**) (31.25 µg/mL), (**C**) (62.5 µg/mL), and (**D**) (125 µg/mL) of the CS and CS-Au hydrogels on Day 0. Scale bar = 500 μm.

**Figure 15:**
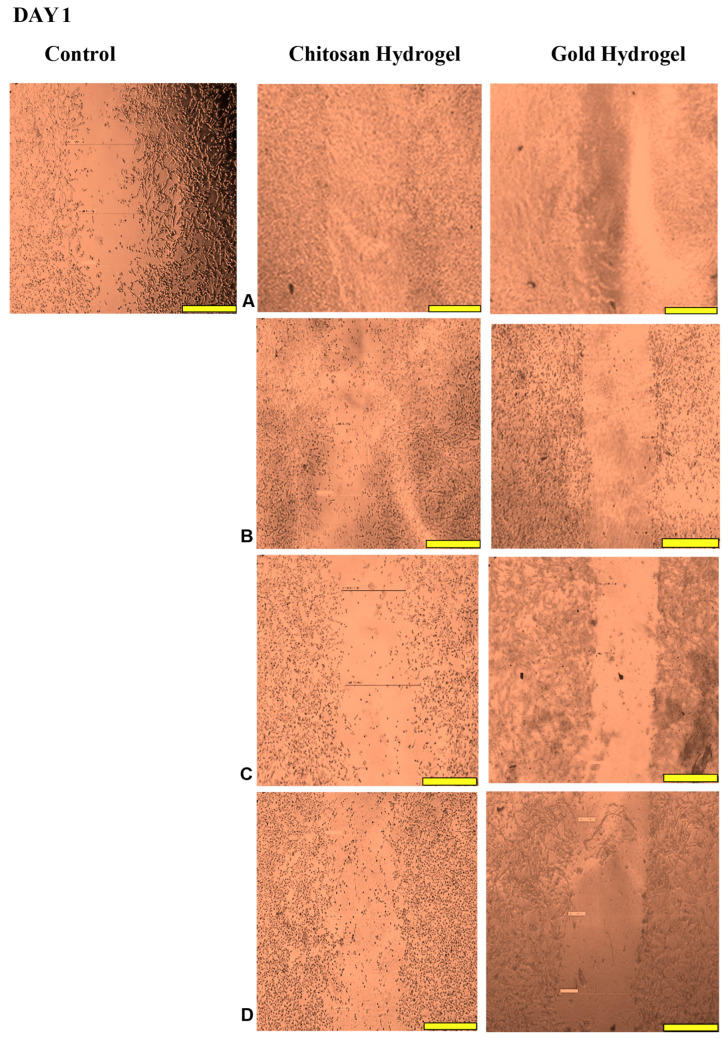
Images displaying the migration of cells for wound closure when treated with (**A**) (15.63 µg/mL), (**B**) (31.25 µg/mL), (**C**) (62.5 µg/mL), and (**D**) (125 µg/mL) of the CS and CS-Au hydrogels on Day 1. Scale bar = 500 μm.

**Figure 16:**
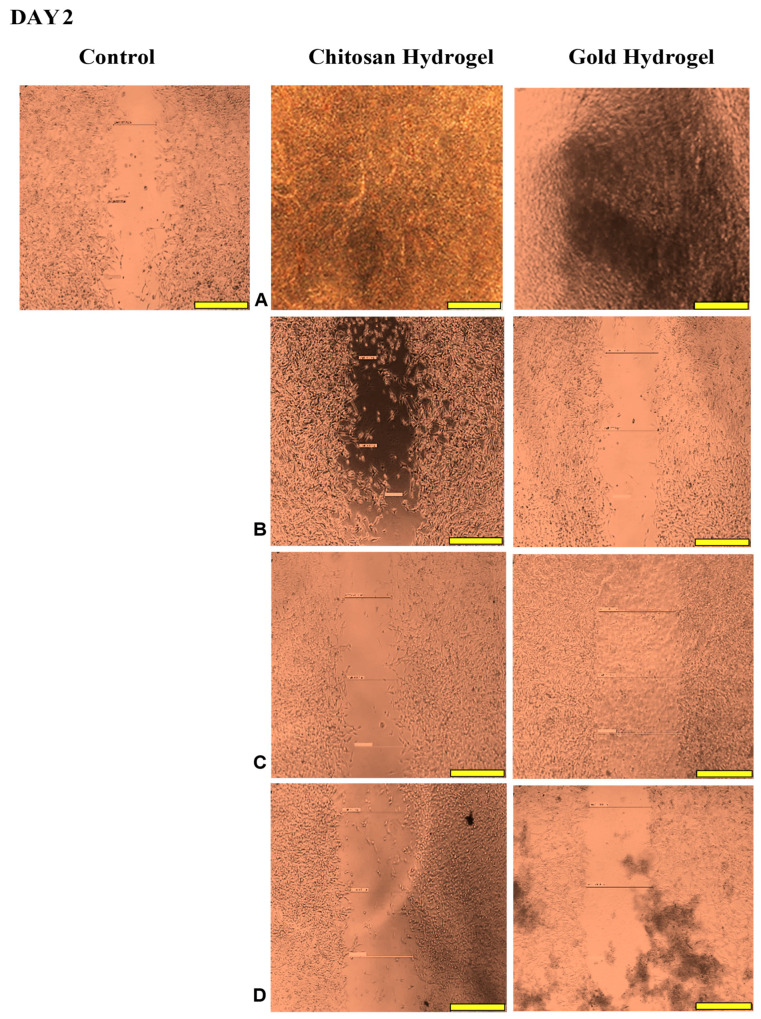
Images displaying the migration of cells for wound closure when treated with (**A**) (15.63 µg/mL), (**B**) (31.25 µg/mL), (**C**) (62.5 µg/mL), and (**D**) (125 µg/mL) of the CS and CS-Au hydrogels on Day 2. Scale bar = 500 μm.

**Figure 17:**
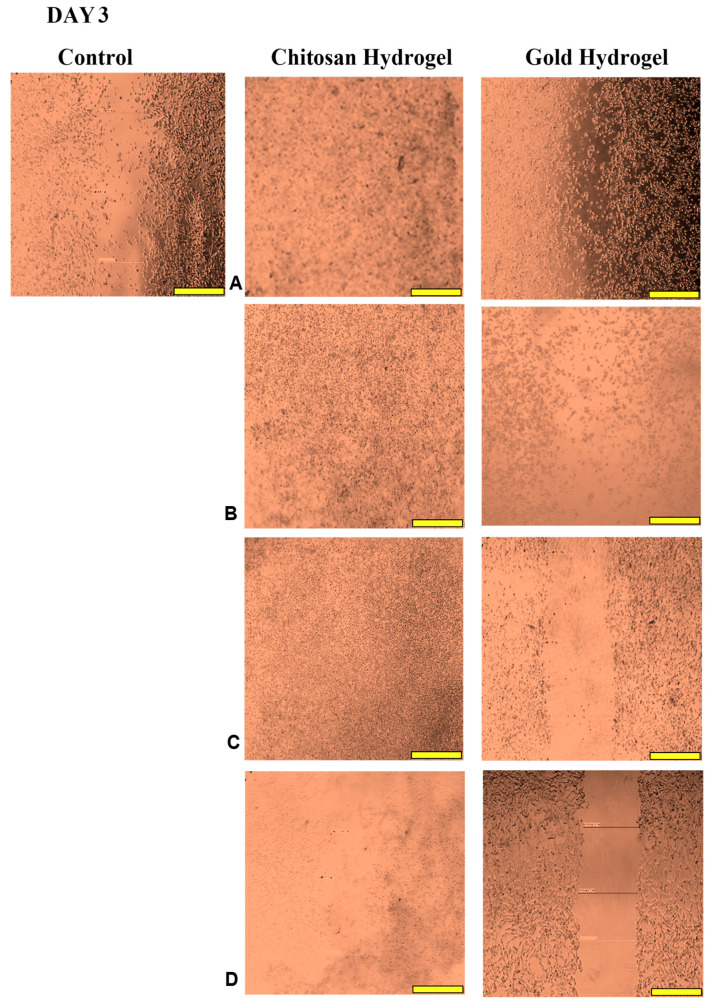
Images displaying the migration of cells for wound closure when treated with (**A**) (15.63 µg/mL), (**B**) (31.25 µg/mL), (**C**) (62.5 µg/mL), and (**D**) (125 µg/mL) of the CS and CS-Au hydrogels on Day 3. Scale bar = 500 μm.

This study examined the migration of human HEK293 cells (normal cell model) following treatment with the CS and CS-Au hydrogels for 3 days. Figure 14, Figure 15, Figure 16 and Figure 17 (Day 0 to Day 3) illustrate that the migratory distance of cells treated with the CS and CS-Au hydrogels exceeded that of the control group, exhibiting a dose-dependent pattern. All concentrations of both hydrogel treatments demonstrated wound closure properties and may be considered appropriate for wound healing (Table 3 and Table 4). Confirmation is established through the gradual migration of cells toward the center of the scratched surface. This arises from the hydrogels mimicking the ECM, which supplies cells with a microenvironment that more precisely reflects the dynamic interdependence between cells and their surroundings. Nonetheless, the treatment efficacy is predominantly observed at the lower dosages of 15.63 (µg/mL) and 31.25 (µg/mL). At 15.63 µg/mL, both the CS and CS-Au hydrogels achieved full wound closure on Day 1. This parallels the MTT experiment, where cell proliferation was observed at a concentration of 20 µg/mL. At a dosage of 31.25 (µg/mL), both treatments achieved complete wound closure, albeit at a diminished rate, as completion was noted only after Day 3. At higher doses (62.5 µg/mL and 125 µg/mL), a decrease in the effectiveness of the CS-Au hydrogel is apparent. The CS hydrogel illustrates complete wound closure by Day 3 at a dose of 62.5 µg/mL, exhibiting a similar response to that observed at 31.25 µg/mL. At a concentration of 125 (µg/mL), efficacy declined significantly, with wound closure reaching just 30.23% on Day 3. CS displayed a comparable wound closure to the control, which had a closure rate of 32.09% after Day 3. Therefore, treatment at 125 µg/mL with the CS hydrogel may not hinder the wound healing process but rather prove ineffectual. Conversely, the CS-Au hydrogel may be considered disruptive to the wound healing process at higher doses (62.5 µg/mL and 125 µg/mL), as it demonstrated wound closure percentages of 21.89% and 20% following Day 3. The wound closure percentages are inferior to the control (32.09%) and may be considered detrimental to the wound healing process, owing to possible AuNP toxicity through temporary stability and possible aggregation [23].

## 4. Discussion

The CS hydrogel and the incorporation of AuNPs were successfully demonstrated using spectroscopic techniques. For the incorporation of the AuNPs, heating resulted in more AuCl_4_^−^ ions being converted to zerovalent Au^0^ and deposited onto the existing Au domains, leading to their growth and aggregation. NP aggregation diminishes the number of free particles in solution, resulting in a reduction in absorbance intensity as fewer particles transmit to the plasmonic phenomena that facilitate absorbance in UV-vis spectroscopy [24]. FTIR is a well-established characterization technique and was successfully employed to verify the functional groups present in the hydrogel.

The zeta potential (ZP) analyzed the electrostatic properties of the system and assessed the rate of NP aggregation, indicating the physical stability of the system [25]. The magnitude of the ZP indicates the repulsive interactions between suspended particles and may be employed to predict the affinity of nanogels for cancer cells. NP stability in the context of biological systems poses a significant challenge for therapeutic application due to the high surface area-to-volume ratio [26]. Strongly negative or positive zeta potential values generate significant repulsive forces, thus inhibiting aggregation and facilitating effortless redispersion. A zeta potential greater than ±30 mV is preferable for physical stability [27]. Furthermore, due to its protonated amino groups, C S yields a positive ZP. The positive charge may enhance drug delivery due to the electrostatic interaction between negatively charged cancer cells and the positively charged hydrogels.

Due to its dense core, gold scatters the electron beam from the TEM and appears as black patches on a bright background [28,29]. Cellular uptake and drug release directly correspond to the dimensions of the NPs, offering a glimpse at the potential effectiveness of the CS-Au hydrogel. It has been demonstrated that NPs with a diameter of less than 10 nm are expeditiously excreted by the kidneys, but those over 200 nm may trigger the complement system via foreign body clearance mechanisms [30]. The NPs exhibited a dendrimer-like morphology emanating from the attractive forces among the NPs within the gel matrix that facilitate their movement, agglomeration, and crystal formation [18]. This was substantiated by the low ZP values obtained.

The cross-linking matrix of the hydrogel affects its capacity for swelling and viscosity, and its dense structure may influence its mechanical properties, drug encapsulation, and drug delivery. In terms of wound healing, the enhanced rigidity resulting from the cross-linking matrix facilitates durability in dynamic wound surface applications. Upholding structural integrity is essential in ensuring the efficacy of the hydrogel throughout the wound-healing process. The observed aggregation under TEM may diminish the effectiveness of the hydrogel by constraining its accessible surface area for engagement with bacteria, among other components that compose the wound site.

Morphological characterization explicates the diverse physical features of hydrogels, including their hydrophilicity, rigidity, and swelling behavior. The inconsistency in pore size and distribution is a feature of a pure CS hydrogel. Future research can address uniformity by integrating polymers such as polyacrylamide [31]. The porosity of the hydrogels provides the foundational attributes for adequate drug encapsulation and release. The network of interlinked pores also mimics the ECM, establishing a scaffold for tissue restoration. Furthermore, the porous structure may facilitate enhanced oxygen exchange and fluid regulation, which is essential for sustaining the optimal wound environment [32].

The CS-Au hydrogels exhibited a reduced weight loss, attributable to the enhanced stability conferred by their cross-linked structure. Furthermore, the water content in the CS hydrogel is influenced by the material’s quantity and nature of ionic groups [18]. The AuNPs partially occupy these ionic sites (hydroxyl and amino). Hence, the CS-Au hydrogel exhibited decreased weight loss compared to the CS hydrogel due to fewer ionic functional groups, reducing absorbed water. The high stability of the hydrogels at physiological temperature could suggest a potential for sustained and regulated drug release. Regarding wound healing, the hydrogel may be applied for wound closure as it does not significantly degrade in structure and functionality at body temperature. The decomposition of CS at high temperatures entails the disintegration of glycosidic bonds, considerably contributing to weight loss. The electrostatic interactions between the CS and AuNPs were disrupted, resulting in an impairment in thermal stability. The CS and CS-Au hydrogels exhibited rapid weight loss, retaining mere residues post-heating. Incorporating AuNPs into a CS gel has shown an enhancement in thermal stability relative to hydrogels devoid of NPs.

Flow curves (stationary shear flow) delineate the rheological characteristics of the hydrogel, and notably the relationship between viscosity and the applied shear rate. The biomedical application of hydrogels necessitates the ability to exhibit shear-thinning behavior and rapid self-healing capabilities post-injection. Consequently, incorporating physical cross-links through electrostatic interactions between the CS and the AuNPs is essential for transient deformation to facilitate injectability. Shear-thinning (pseudoplastic) hydrogels are regarded as injectable matrices that develop via reversible physical interactions under mild conditions and demonstrate a reduction in viscosity with an increase in shear rate [33]. The shear-thinning property of the synthesized hydrogels facilitates their flow into fissures and close conformation to the wound bed, thereby enabling full coverage and treatment of the entire surface [34]. This also reduces mechanical damage to sensitive regenerating tissues following application or patient movement. By minimizing the possibility of disrupting new tissue, this subtle contact with the wound site helps to promote rapid and less painful healing.

Both the CS and CS-Au hydrogels demonstrated self-healing properties. The CS-Au hydrogel retained its shear-thinning and self-healing characteristics, which can be attributed to its CS hydrogel precursor. This is due to the relatively weak electrostatic interactions between the CS and the AuNPs [35]. The reversion to a solid gel is clinically essential, as the integrated 5-FU can be administered to the injection site and remain localized as the hydrogel regenerates. The self-healing capability in wound healing permits the reapplication or modification of the hydrogel without undermining its efficacy. It also enhances the long-term durability of the dressing, decreasing the necessity for frequent replacements and minimizing any disruption to the wound.

During the re-swelling process, water molecules infiltrate the hydrogel, inducing the hydrogel network to expand due to the hydration and unwinding of the CS chains. The reversible water absorption capacity demonstrated that the CS-Au hydrogel possesses substantial mechanical strength and stability in aqueous solutions. Furthermore, CS exhibits considerable hydrophilicity, which significantly increases the water absorbency of the hydrogel [36]. The rapid re-swelling rate of the CS-Au hydrogel can be attributed to its porous interconnecting framework, which offers many ways for the diffusion of water molecules. This extensive swelling further substantiates the morphological analysis. The high absorption of water is due to the protonation of the amino groups of CS, which increases the mesh size of the cross-linking network. Protonation enhances the hydrophilic capability of the CS polymer and its tendency to interact with water molecules, resulting in more significant swelling. The protonation of amino groups also increases the positive charges of the polymer chains. The repulsion among the positive charges causes the polymer chains to broaden, culminating in the widening of the hydrogel pores, which increases water absorption and swelling [37]. At neutral or basic pH, amino groups are deprotonated, reducing swelling.

The pH sensitivity and elevated water absorption are promising for drug release, as the enhanced water influx and pore expansion at acidic pH may facilitate a better diffusion of 5-FU in an acidic cancer microenvironment than at physiological pH (healthy cells). Regarding wound healing, the hydrogel may also absorb significant amounts of wound exudate due to its high swelling capacity, which also acts to retain moisture. This is important for facilitating cell growth and migration, which are crucial for wound healing. A moist wound environment promotes healing by minimizing tissue dehydration and scab formation, which hinders healing. Wound exudate management also inhibits bacterial proliferation and infection, which is paramount in preventing the stagnation of the healing process [38]. 

A favorable drug encapsulation efficiency was achieved, comparable to AuNP formulations in the literature [39,40]. Cross-linking density reduction can enhance porosity and hydrogel swelling, leading to increased drug encapsulation [41]. Furthermore, a decrease in the size of AuNPs and aggregation can contribute to reduced density and increased drug absorption. 

Lysozyme is present in several tissues and fluids in the human body. Blood serum and wound exudates contain lysozyme at concentrations around 1.5 µg/mL, which breaks down hydrogels used in drug delivery and as wound dressings during the healing process [42]. According to the morphological analysis, the hydrogels displayed a porous interconnecting network, which is vital for enzyme transport and the degradation of products crucial for tissue repair. The degree of deacetylation of the CS (75%) used allows for the action of the lysozyme due to its complexation with the acetyl groups. Lysozyme primarily targets the glycosidic linkages in CS, which are characterized by β (1 → 4) links between the glucosamine units. Upon interaction with the hydrogel, lysozyme catalyzes the hydrolysis of these bonds, degrading CS into glucosamine units, which are readily metabolized or expelled by the body [43]. Lysozyme-facilitated degradation is advantageous as the byproducts can enhance the regeneration and repair of tissues [44]. It also guarantees that the hydrogel does not require surgical removal following drug delivery, as it will disintegrate naturally over time. The faster breakdown of the CS-Au-5-FU hydrogel may be attributed to the encapsulation of 5-FU, which is noted for its ionic complexation with CS chains [45].

During drug release, an initial quick-release or “burst effect” occurred due to the quantities of 5-FU localized on the hydrogel surface via adsorption, allowing rapid release through diffusion [46]. The diffusion of proteins, NPs, or drugs within hydrogels depends on the pores formed by the cross-linking of polymer chains. The hydrogel mesh size is significantly influenced by pH and ionic strength due to the protonation or deprotonation of CS, as seen in the reswelling and pH sensitivity assay. At acidic pH, the relaxation and protonation of the CS polymer chains result in an enlargement of the pore size within the cross-linking matrix, subsequently enhancing swelling and accelerating diffusion [47]. This study demonstrated a sustained drug release pattern, which allows for the continuous exposure of anticancer agents to the cancer tissue. Continuous release is essential for minimizing large dosage regimens, which is a disadvantage in drug administration [48]. A diminished release at a neutral pH is advantageous to reduce damage to healthy tissue. The prolonged-release profile holds promise for wound healing investigations that involve the delivery of antibacterial or anti-inflammatory drugs, as it can reduce the need for reapplication and improve the durability of each therapy.

The objective of a drug release system is to sustain the quantity of the therapeutic in the bloodstream or target tissues at the desired level for an extended period. A controlled-release device can initially discharge a portion of the dose to swiftly achieve the effective therapeutic dosage of the drug (burst release). Well-defined drug release kinetics can help maintain an efficient drug concentration level [49]. Based on the release profile demonstrated, the CS-Au-5-FU hydrogel may be a suitable drug delivery system. The drug release rate of a polymer-based delivery system is affected by diffusion, swelling, erosion, and the surrounding environment [50]. The equilibrium of absorption on the exposed surface of the polymeric system occurs swiftly, resulting in linear time-dependent release conditions [51]. The kinetics are defined by diffusivity, facilitating more predictable and regulated drug distribution. This allows for the design of systems that consistently release drugs over time. 

The minimal cytotoxicity of the hydrogels suggests their effectiveness in wound healing, which was further clarified in the scratch assay. The CS-Au-5-FU hydrogel exhibited cytotoxicity in the HEK293 cells, as anticipated from the drug release profile. At pH 7.4, which is associated with healthy cells such as HEK293, 33.41% of 5-FU was released from the nanocomplex after 48 h, leading to reduced cell viability. The treatment of HEK293 with the nanocomplex achieved greater cell viability compared to free 5-FU, indicating the superiority of the nanocomplex over the free drug in cancer therapy. Research suggests that non-cancer cells, with their neutral or slightly positive charge from buffer systems and ion channels, contribute to increased cell viability by reducing their attraction to positively charged hydrogels [52]. The nanocomplex possessed a positive zeta potential, which may have added to the reduced cytotoxicity.

The favorable cytotoxicity results can be attributed to the strong affinity of the nanocomplex for the negatively charged cancer cell membranes in conjunction with the controlled release and retention of 5-FU due to the cationic CS, which augments the swelling and drug diffusion in the acidic cancer microenvironment. CS has also been noted to protect drugs from harsh in vivo conditions and inhibit degradation, compared to free drugs that are susceptible to breakdown [53]. CS, due to its biocompatibility, has been shown to increase the proliferation of cells in vitro [12]. The introduction of cross-linked AuNPs in the cationic CS hydrogel positively impacted the cytotoxicity in the cancer cells, as they penetrated cell membranes due to their diminutive size and synergistically acted with the drug payload. Thus, the CS-Au-5-FU hydrogel may decrease dosage regimens and mitigate the adverse effects of prolonged treatment.

The wound healing studies showed that cells from the edges of the wound proliferate and migrate into the center of the site of injury, facilitating re-epithelialization of the surface and restoring the skin’s barrier function. The promotion of migration is key for an optimal wound dressing material. Scratch assays are frequently employed to examine the in vitro movement of cells in response to diverse stimuli. The ECM influences cell migration in terms of both the temporal and tension scales of cell-ECM interactions, as cells detect their environment via their membrane and adapt by reorganizing cytoskeletal components [54]. The ECM functions as a substrate for cellular adhesion, enabling cell attachment, spreading, and migration [55]. The mechanical properties of hydrogels can also influence the capacity of cells to exert traction forces, thereby impacting migration velocity and/or mode [56]. The disparities in cell migration between the CS and CS-Au hydrogels arise from the mechanical matrix features. The interplay between the intrinsic contractility of the cell and the ECM stiffness influences cell adhesion characteristics [54]. Cell migration is faster in softer gels and impeded in stiffer ones [56], substantiating the results achieved. Integrating AuNPs as cross-linkers is crucial to ensure the stability and water retention required for cellular control and application in vivo. During treatment for wound healing under adverse conditions, such as bacterial presence, the AuNPs may prove more suitable, as gold nanocarriers can adhere to bacterial membranes, causing the release of bacterial components and resulting in bacterial cell death [12]. The findings of the scratch assay suggest that both hydrogels at reduced concentrations facilitated the migration of cells to the injury site and triggered the proliferative phase of wound healing, as evidenced by the complete closure of the cell monolayer. 

## 5. Conclusions

To address deficiencies in drug delivery and wound healing, an innovative hybrid CS-Au hydrogel was synthesized by reducing HAuCl_4_ in the acidic CS-acrylic acid hydrogel, as verified by UV-vis spectroscopy, FTIR, and TEM analysis. The hydrogel exhibited an irregular porous structure and an elevated pH-dependent water absorption capacity, facilitating the diffusion of 5-FU in cancer microenvironments and emulating the ECM for wound healing. The CS-Au hydrogel exhibited high drug encapsulation, enabling effective cellular uptake for enhanced in vitro cytotoxicity. This study offers substantial evidence supporting the approach that prolonged pH-dependent drug release from the CS-Au-5-FU hydrogel results in localized cytotoxicity in cancer. This was supported by the cytotoxicity assay, which indicated that the CS-Au-5-FU hydrogel displayed selective anticancer efficacy in the HeLa and MCF-7 cells compared to the free drug, suggesting the feasibility of using lower dosage regimens and reduced drug concentrations. The wound-healing properties of the CS and CS-Au hydrogels demonstrated complete wound closure at low concentrations, indicating the effective simulation of the ECM. Overall, the synthesized hydrogels have demonstrated the potential for use as future drug delivery systems in cancer therapy and scaffolds for wound healing applications. This opens up new avenues in drug delivery and wound healing to explore and necessitates further optimizations for in vivo applications.

## Figures and Tables

**Table 1:** Zeta potential values of the CS-Au and CS-Au-5-FU hydrogels.

Hydrogel	Zeta Potential (mV)
CS-Au	+11.10 ± 0.1
CS-Au-5-FU	+15.87 ± 1.18

**Table 2:** Kinetic parameters at pH 4.5, 6.5, and 7.4, where (r)^2^ is the coefficient and n^(a)^ is the Korsmeyer–Peppas release exponent.

pH	Zero (r)^2^	First (r)^2^	Korsmeyer–Peppas (r)^2^	n^(a)^
4.5	0.9314	0.8854	0.9959	0.2797
6.5	0.9384	0.9308	0.9671	0.3168
7.4	0.9428	0.9329	0.9759	0.2442

**Table 3:** Wound closure (%) after treatment with various concentrations of the CS hydrogel over 3 days.

Days	% Wound Closure at Different CS Hydrogel Concentrations			
	15.63 (µg/mL)	31.25 (µg/mL)	62.5 (µg/mL)	125 (µg/mL)
Day 1	100%	25.97%	9.95%	2.97%
Day 2	100%	46.25%	40.90%	13.74%
Day 3	100%	100%	100%	30.23%

**Table 4:** Wound closure (%) after treatment with various concentrations of the CS-Au hydrogel over 3 days.

Days	% Wound Closure at Different CS-Au Hydrogel Concentrations			
	15.63 (µg/mL)	31.25 (µg/mL)	62.5 (µg/mL)	125 (µg/mL)
Day 1	100%	21.87%	7.79%	4.75%
Day 2	100%	32.62%	9.46%	14.82%
Day 3	100%	100%	21.89%	20%

## Data Availability

All data presented in the study are included in the article. Further inquiries can be directed to the corresponding author.

## Abbreviations

The following abbreviations are used in this manuscript:

ATR	Attenuated total reflectance
AuNPs	Gold nanoparticles
CS	Chitosan
DLS	Dynamic light scattering
DMSO	Dimethyl sulfoxide
DSC	Differential scanning calorimetry
ECM	Extracellular matrix
EMEM	Eagle’s minimum essential medium
FBS	Fetal bovine serum
FTIR	Fourier transform infrared
5-FU	5-fluorouracil
HEK293	Human embryonic kidney cells
HeLa	Human cervical carcinoma cells
MCF-7	Human breast adenocarcinoma
SEM	Scanning electron microscopy
SPR	Surface plasmon resonance
SR	Swelling ratio
TEM	Transmission electron microscopy
TGA	Thermogravimetric analysis
TME	Tumor microenvironment
UV-vis	Ultraviolet-visible
ZP	Zeta potential
